# Simulation of the Service Environment and Selection of the Refractory Lining for a Heat Recovery Coke Oven

**DOI:** 10.3390/ma17071565

**Published:** 2024-03-29

**Authors:** Yuansheng Zhou, Lixin Zhang, Enhui Wang, Enxia Xu, Zhijun He, Tao Yang, Xinmei Hou

**Affiliations:** 1 Henan Key Laboratory of High Temperature Functional Ceramics, School of Materials Science and Engineering, Zhengzhou University, Zhengzhou 450052, China; zhouyuanshengzzu@163.com (Y.Z.); xexia@zzu.edu.cn (E.X.); 2 Sinosteel Luonai Materials Technology Corporation, Luoyang 471000, China; lynhzlx@163.com; 3 Institute for Carbon Neutrality, University of Science and Technology Beijing, Beijing 100083, China; houxinmeiustb@ustb.edu.cn; 4 Liaoning Academy of Materials, Shenyang 110167, China; 5 Key Laboratory of Green Low-Carbon and Intelligent Metallurgy Liaoning Province, University of Science and Technology Liaoning, Anshan 114051, China; hzhj2002@126.com

**Keywords:** heat recovery coke oven, numerical simulation, silica bricks, reference, thermal stress

## Abstract

A heat recovery coke oven (HRCO) is one of important approaches to achieving a carbon peak and carbon neutrality in China. However, the steady operation of an HRCO is significantly influenced by the internal working conditions and the quality of lining refractories. In this work, a comprehensive study of the internal working conditions of an HRCO was carried out. The results suggest that the partition wall (PW) between the carbonization and combustion chambers is the most vulnerable area, with the corresponding traditional silica bricks inadequate for the service requirements. A reference based on a comparison of the average thermal stress and high-temperature compressive strength is offered for evaluating and selecting silica bricks for the PW. New optimized silica bricks within the reference are verified to be more applicable to the actual working conditions of an HRCO than the traditional silica bricks. As such, this work provides valuable guidance for the optimization and selection of silica bricks for the PW in an HRCO.

## 1. Introduction

Coke, as an important raw material in the metallurgical and chemical industry, is mainly produced via coke ovens. The primary function of coke ovens is to transform coal into coke through a pyrolysis process. However, this process not only extracts volatile constituents and augments the carbon content of the coal but also leads to emissions of particulate matter, sulfides, nitrogen oxides and volatile organic compounds, adversely affecting the air quality [1,2,3]. Given the goals of carbon peaking and carbon neutrality in China, the environmental pollution caused by traditional coke ovens is increasingly highlighted [4,5,6]. To solve this issue, a new heat recovery coke oven (HRCO) has been proposed recently. Compared with a traditional coke oven, an HRCO replaces the traditional coke oven’s battery (dozens of carbonization chambers connected with combustion chambers interactively) with a large-volume carbonization chamber and combustion chamber [7,8,9,10]. In addition, the operating condition is adjusted to a negative pressure so that low emissions and low pollution can be realized [11]. Nevertheless, the high temperature at the top of the furnace wall of the combustion chamber and the large temperature difference at the bottom of the furnace wall of the carbonization chamber lead to the degradation of silica bricks used in the partition wall (PW) in an HRCO. The resulting cracks and collapse of silica bricks can seriously affect the steady operation of an HRCO and even cause industrial accidents [12,13,14,15]. At present, the internal working conditions of an HRCO have not been clearly studied, which greatly obstructs the material selection and performance improvement of silica bricks.

Since the internal working conditions in an HRCO are complex, numerical simulation offers an efficient and feasible method for studying these conditions. The existing research is mainly focused on traditional coke ovens. For example, Xiao et al. [16] adopted a multi-chamber coupling mathematical model to simulate the temperature variations and distributions in a coke oven when using silica bricks with different thermal conductivities. Farias et al. [17], meanwhile, studied the combustion chamber temperature under different coke oven gas contents and obtained several temperature profiles. Based on this, they proposed a feasible approach to improve the heat transfer efficiency in the coke oven. In another study, Smolka et al. [18] used a validated transient coupled model to determine the thermal parameters that occur on both sides of the heating wall in the coke oven battery. The obtained information was then employed to regulate the heating subsystem in a coke oven battery in terms of efficient heat delivery and prediction of the coking cycle’s completion. These studies suggest the validity of numerical simulation for studying the internal working conditions of traditional coke ovens. However, the characteristics of the internal working conditions of an HRCO remain unclear owing to the significant differences in working temperature and pressure, as well as oven structure, when compared with a traditional coke oven.

In that context, this article aims to provide guidance on the material selection of silica bricks by clarifying the internal working conditions of an HRCO. Firstly, the temperature, flow, pressure and stress fields inside an HRCO are simulated with the aid of the Ansys Fluent software (the software version is Ansys 2021) [19]. Secondly, the simulation results are extracted as a reference for selecting silica bricks for an HRCO. Finally, the validity of the reference is evaluated by comparing the performance of the traditional and optimized silica bricks through simulation and experimental approaches under actual working conditions. Ultimately, valuable guidance is offered on the optimization and selection of silica bricks to be applied in the PW of an HRCO, which will contribute to the realization of carbon peaking and carbon neutrality goals.

## 2. Methods of Numerical Simulation

### 2.1. Model of Geometry

**Figure 1:**
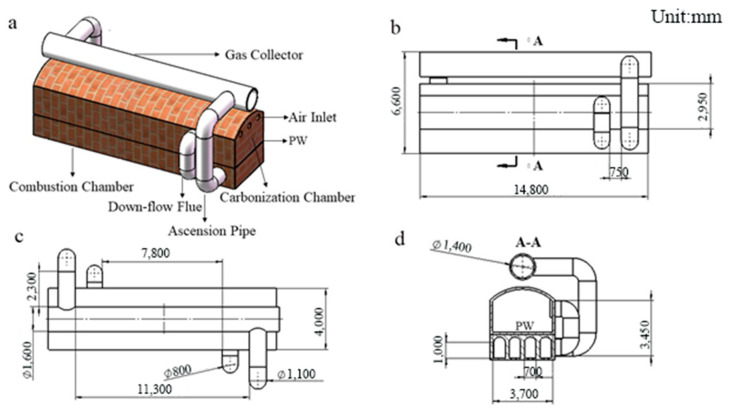
The structural model of an HRCO: (**a**) 3D model, (**b**) front view, (**c**) top view, (**d**) side view.

**Figure 2:**
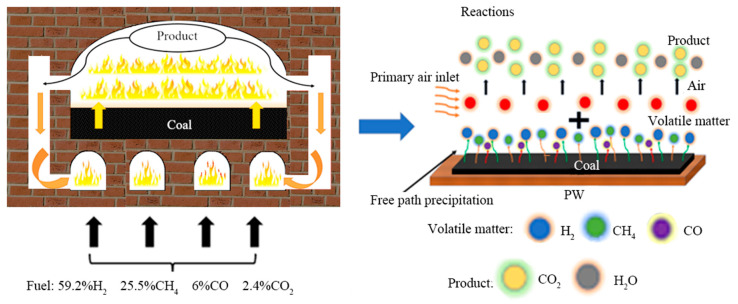
The internal reactions of an HRCO.

A model of an HRCO was constructed, as shown in Figure 1, in which the total height, length and width were set to 6 m, 14.8 m and 4 m, respectively [20]. From Figure 2, it can be seen that the heat is transferred from the combustion chamber to the carbonization chamber via the PW during the operation of a heat recovery coke oven (HRCO). Then, the volatiles produced by high-temperature pyrolysis of coal enter the fire channel of the combustion chamber along the down-flow flue. Finally, the exhaust gas flow discharges through the gas collector along the ascension pipe [21]. Based on this setup, some assumptions regarding the production process are herein proposed. 

### 2.2. Governing Equation

The internal working conditions of an HRCO involve incompressible homogeneous fluid and a constant density, so the following models in Table 1 are suitable for an HRCO.

(1) Turbulence model

This model involves the mass conservation equation, the momentum conservation equation, and the energy conservation equation. Besides, the standard k-Ꜫ model is set as the turbulence equation (turbulent momentum equation and turbulent energy equation).

(2) Heat transfer model

Heat transfer includes the convection heat transfer model and radiative heat transfer model. Considering that an HRCO has a high internal temperature and 90% radiative heat dissipation, the P1 model is set as the radiative heat transfer model [22].

### 2.3. Boundary Conditions and Parameters

In the combustion chamber, the boundary condition for fuel entry is set as a velocity inlet. Meanwhile, in the carbonization chamber, the boundary condition for coal volatiles is set as a mass flow inlet. Additionally, the boundary conditions for both primary and secondary air inlets are set as velocity inlets. The boundary condition parameters are set as shown in Table 2.

### 2.4. Assumptions and Solution Methods

The following assumptions are introduced during the simulation process.

a. The reaction of volatiles with air is set as a standard finite rate model.
b. The heat transfer coefficient between the fluid inside the HRCO and the lining is constant.
c. The chemical reaction of volatiles with air in the carbonization chamber is not taken into account in the volatiles’ precipitation route.
d. The gases like methane (CH_4_), hydrogen (H_2_), carbon monoxide (CO) and water vapor (H_2_O) are released during the coal pyrolysis process at a high temperature. These gases are assumed to move in a mass flow from the bottom to the center of the carbonization chamber.
e. The gas is set as incompressible ideal gas.
f. The exterior of the coke oven is characterized as a gray body, with its emissivity considered as a constant value.

Ansys Workbench was used to analyze the internal flow field and thermal stress field of an HRCO. Firstly, the mesh was divided by the mesh module in Ansys Workbench (the grid orthogonal quality by 10^−3^) and the fluid region was set as the calculation region by a pressure-based solver. Secondly, the gas combustion model [23] was adopted to simulate the fluid flow and combustion reaction. Thirdly, the steady flow field was transmitted to a steady temperature field, in which the transient temperature field [10], fluid pressure and temperature were loaded in the HRCO’s lining and PW. Finally, the thermal stress and deformation were calculated by numerical simulations. 

## 3. Results and Discussion 

### 3.1. The Internal Working Condition of an HRCO

#### 3.1.1. The Internal Working Temperature of an HRCO

**Figure 3:**
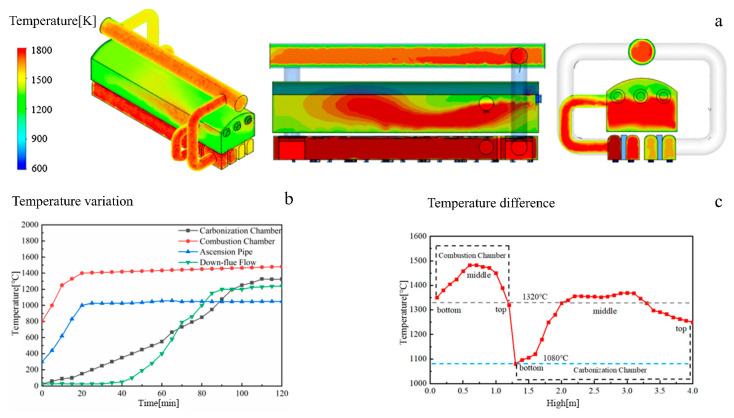
The internal working temperature of an HRCO: (**a**) temperature field, (**b**) temperature variation, (**c**) temperature difference.

The internal working temperature of an HRCO during its steady operation is shown in Figure 3. From Figure 3a, it can be seen that the temperature of the HRCO is between 1200 K and 1800 K, in which the combustion chamber and the carbonization chamber can reach up to 1800 K and 1720 K. In addition, it should be noted that the PW has a large temperature difference, i.e., the temperature at the bottom of carbonization chamber is lower (1300 K) while the temperature at the top of combustion chamber is higher (1700 K). As illustrated in Figure 3b, there is a notable variation in temperature across different internal sections of the HRCO. In addition, a significant temperature difference between the combustion chamber and the carbonization chamber can be observed. Figure 3c reveals that the variation in temperature across different sections inside the HRCO is closely related to the height. In particular, a substantial temperature difference of the PW in an HRCO positioned between the carbonization chamber and combustion chamber can be intuitively observed. Hence, it is reasonable to conclude that the PW faces relatively harsh working conditions, i.e., elevated temperatures along with a pronounced temperature difference.

#### 3.1.2. The Internal Working Pressure of an HRCO

**Figure 4:**
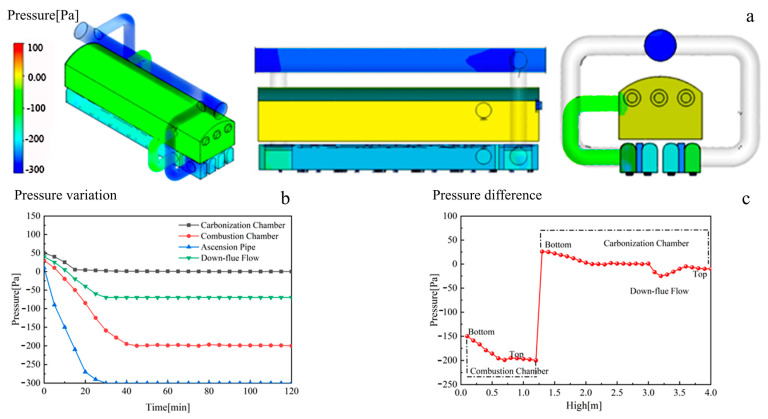
The internal working pressure of an HRCO: (**a**) pressure field, (**b**) pressure variation, (**c**) pressure difference.

The pressure field of an HRCO, as shown in Figure 4a, falls between −300 Pa and 100 Pa. The combustion chamber and the carbonization chamber can reach −100 Pa and 50 Pa, respectively. Therefore, the pressure difference between the two sides of the PW is relatively slight during the steady operation of an HRCO. As illustrated in Figure 4b, there is a notable variation in pressure across different internal sections of an HRCO. Figure 4b demonstrates that during the operation of an HRCO, the internal pressure progressively decreases. Meanwhile, Figure 4c displays an obvious variation in pressure across different sections inside the HRCO with the height increasing. In this context, a lower pressure difference between the carbonization chamber and the combustion chamber exists. Hence, it can be concluded that the pressure on the PW in an HRCO is comparatively low, indicating that the influence of pressure on the silica bricks in a PW is negligible.

#### 3.1.3. The Internal Gas Velocity of an HRCO

**Figure 5:**
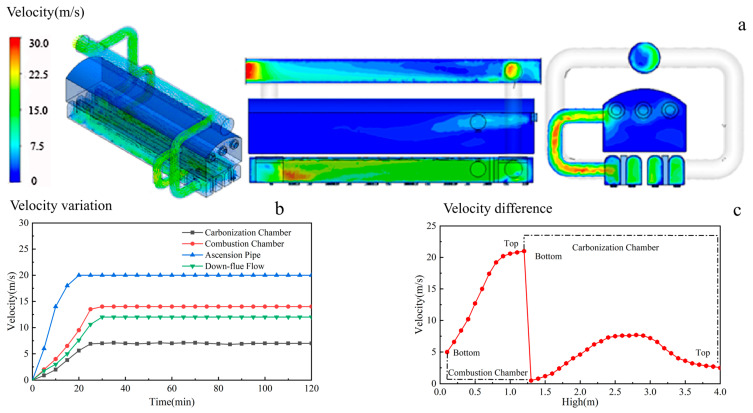
The internal gas velocity of an HRCO: (**a**) velocity field, (**b**) velocity variation, (**c**) velocity difference.

The gas velocity field of an HRCO is shown in Figure 5a, where it can be seen that the flow field of an HRCO from the carbonization chamber successively flows through the down-flow flue, combustion chamber and gas collector. The flow velocity is between 0 m/s and 30 m/s in the HRCO, in which the carbonization chamber and the combustion chamber can reach up to 6 m/s and 25 m/s, respectively. Consequently, during the operation of the HRCO, there is a markedly higher flow velocity in the vicinity of the PW. Figure 5b presents the changes in gas flow velocities within different regions of an HRCO over time. From Figure 5b, it can be found that the gas flow velocity near the PW is consistently high. Figure 5c illustrates an obvious variation in velocity across different sections inside an HRCO with the height increasing, where a substantial velocity difference between the carbonization chamber and the combustion chamber is present. Therefore, it can be inferred that the gas flow velocity near the PW is relatively high.

#### 3.1.4. The Internal Thermal Stress of an HRCO

**Figure 6:**
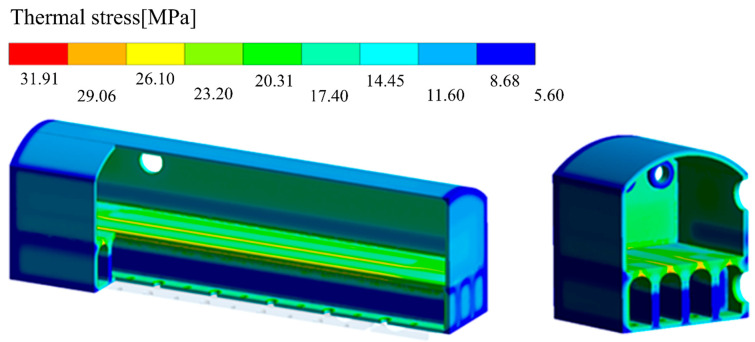
The internal thermal stress of an HRCO.

An HRCO’s thermal stress is shown in Figure 6, where the PW (marked by the darkest color) exhibits thermal stress ranging from 20.31 MPa to 26.10 MPa, with an average of approximately 23.20 MPa. In comparison, the thermal stress in other parts of the HRCO is relatively lower, ranging from 5.60 MPa to 20.31 MPa. Based on the above simulation results, the PW is inferred to be the most vulnerable area since it is exposed to extreme conditions including a high temperature difference, significant thermal stress and high flow velocity in an HRCO. In this context, the silica bricks used at present are unfavorable for the long and stable operation of an HRCO. Therefore, developing and optimizing new types of silica bricks to address these challenges are urgent requirements.

### 3.2. Proposal of a Reference for Evaluating Silica Bricks

**Figure 7:**
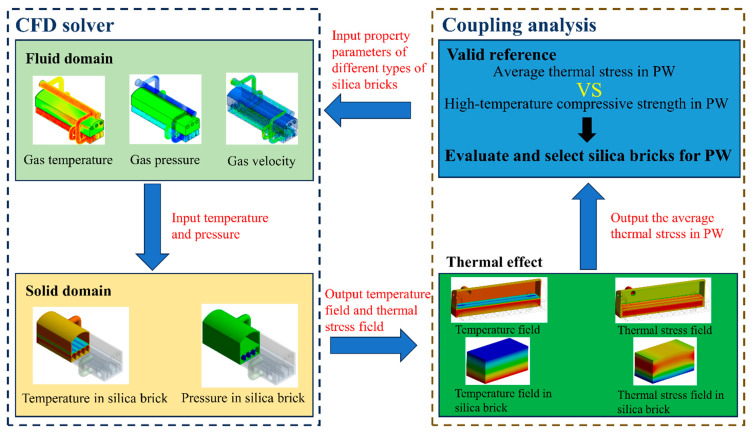
Solution process of the valid reference.

The thermal stress and thermal deformation within silica bricks in the PW are primarily influenced by the thermal expansion coefficient, Young’s modulus and thermal conductivity. In order to propose a valid reference for evaluating the feasibility of silica bricks, a fluid–solid thermal coupling method, as shown in Figure 7, was employed to simulate the impact of these factors on the performance of silica bricks in the PW under actual working condition, as provided in the Section 3.1. For different types of silica bricks, in Table 3, theoretical values are given for simulations, which are selected from the specific range of thermal conductivity, Young’s modulus and thermal expansion coefficient for common silica bricks. These theoretical values are divided into six levels and have an orthogonal design with three factors. In this way, the number of the simulation iterations can be decreased to some extent, improving the simulation efficiency for selecting suitable properties of silica bricks. Based on the fluid–solid thermal coupling simulation, the average thermal stress and top temperature of the PW were calculated for different types of silica bricks with specific property parameters, as listed in Table 3. Under high-temperature conditions, silica bricks tend to experience alterations in their structure and performance, resulting in a decrease in their high-temperature compressive strength. Once the thermal stress is beyond their compressive capacity, cracks, spallation and/or other damage in the bricks will appear. Therefore, it is reasonable to adopt “high-temperature compressive strength vs. average thermal stress” as a criterion for evaluating the feasibility of silica bricks [24,25].

Table 4 presents the comprehensive properties of both traditional and optimized silica bricks. Combined with the results in Table 3, it can be seen that the thermal expansion coefficient, Young’s modulus and thermal conductivity of traditional silica bricks are closely aligned with the data presented in the 14th entry. In comparison, the average high-temperature compressive strength (19.2 MPa) of traditional silica bricks is lower than the average simulated thermal stress (21.80 MPa). Therefore, the traditional silica bricks in the PW are confronted with the issue of insufficient stability. To address this issue, a kind of new optimized silica brick has been developed, as shown in Table 4. The comprehensive properties of this optimized silica brick are closer to the 23rd entry in Table 3, in which the average thermal stress is calculated to be 24.86 MPa. Considering that the average high-temperature compressive strength is 36.6 MPa, the optimized silica bricks should be more applicable to the service requirements of the PW in an HRCO than the traditional silica bricks.

### 3.3. Evaluation of the Feasibility of the Reference

**Figure 8:**
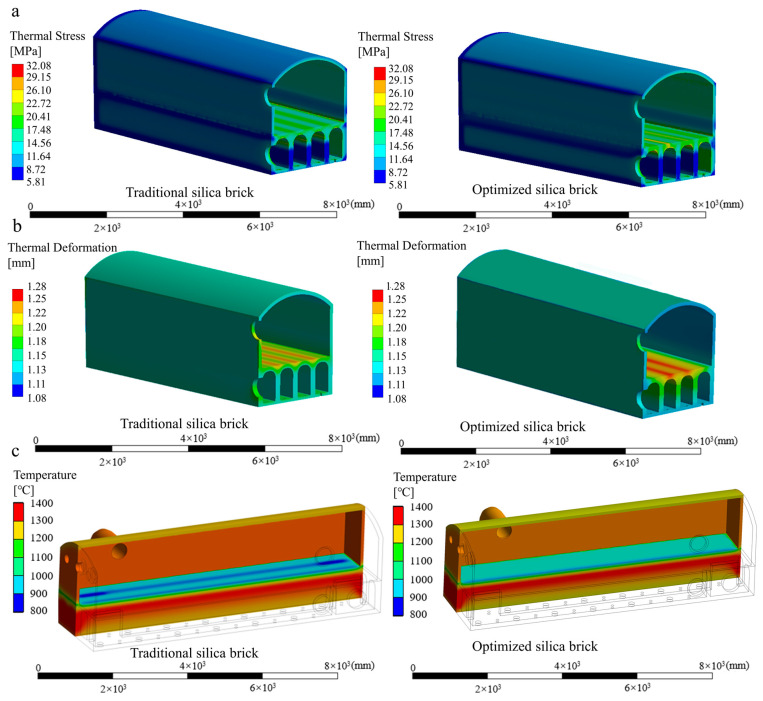
The internal working conditions of an HRCO applied to the two types of silica bricks: (**a**) thermal stress, (**b**) thermal deformation, (**c**) temperature.

To evaluate the feasibility of this reference, a simulation method and an experimental method (based on monitoring data collected during industrial production) were employed to compare the two types of silica bricks under actual working conditions, as provided in Section 3.1. For simulation verification, the thermal stress, thermal deformation and temperature experienced by both traditional and optimized silica bricks in the PW were calculated, as shown in Figure 8. From Figure 8a, it can be found that the thermal stress of traditional silica bricks is between 17.48 MPa and 26.10 MPa, while the thermal stress of optimized silica bricks ranges from 17.48 MPa to 29.15 MPa. Hence, the optimized silica bricks exhibit a higher average high-temperature compressive strength (36.6 MPa) in comparison with the average thermal stress (26.24 MPa). Nevertheless, the high-temperature compressive strength of optimized silica bricks in localized areas of the PW is lower than the maximum thermal stress. As illustrated in Figure 8b, there is a slight difference in the thermal deformation of optimized silica bricks and traditional silica bricks. From Figure 8c, it can be observed that the temperature of the PW made with optimized silica bricks is more evenly distributed and higher compared to that with traditional silica bricks. Based on our comparison of the actual performances of the two types of silica bricks, as shown in Table 4, it can be concluded that the optimized silica bricks are more applicable to the service requirements. This verifies the validity of the reference from a simulated perspective. 

**Figure 9:**
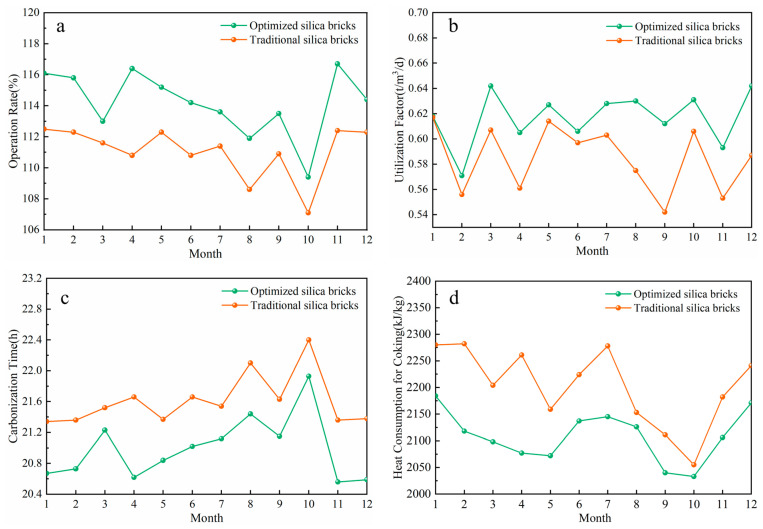
Production data: (**a**) operation rate, (**b**) utilization factor, (**c**) carbonization time, (**d**) heat consumption for coking.

From the point view of practical application, both the optimized and traditional silica bricks were employed in a coke oven factory over a period of time. Figure 9 provides the monitoring data for this practical application of traditional and optimized silica bricks. Figure 9a,b reveal that the operation rate and utilization factor of an HRCO using optimized silica bricks consistently surpass those of another with traditional silica bricks. Conversely, as demonstrated in Figure 9c,d, both the carbonization time and heat consumption when using optimized bricks are lower compared to using traditional ones. These results suggest that the optimized silica bricks are more suitable for the working conditions of an HRCO than the traditional silica bricks, which further verifies the validity of the reference.

## 4. Conclusions

In this work, the internal working conditions of an HRCO have been studied with aid of numerical simulations. The PW between the combustion chamber and carbonization chamber has been found to be most vulnerable area since it is exposed to a large temperature difference (240 K), significant thermal stress (26.10 MPa) and high flow velocity (25 m/s), leading to the unsatisfactory performance of traditional silica bricks. A reference based on our comparison of the average thermal stress and high-temperature compressive strength has been proposed to guide the selection of silica bricks for use in the PW. The optimized silica bricks exhibit a higher average high-temperature compressive strength (36.6 MPa) in comparison with the average thermal stress (26.24 MPa), which demonstrates that they are more applicable than traditional silica bricks to the service requirements of a PW in an HRCO. In the future, research should focus on the further improvement of the comprehensive properties of silica bricks, with the goal of prolonging their service life.

## Figures and Tables

**Table 1:** The equations of the working conditions in an HRCO.

Title	Equation			NO.
Mass conservation equation	$\frac{𝜕\rho }{𝜕t}+\frac{𝜕}{x_{i}}(\rho \mu_{i})=s_{m}$			(1)
Momentum conservation equation	$\frac{𝜕}{𝜕t}(\rho u_{i})+\frac{𝜕}{𝜕x_{i}}(\rho u_{i}u_{j})=-\frac{𝜕p}{𝜕x_{i}}+\frac{𝜕\tau_{ij}}{𝜕x_{i}}+\rho g_{i}+F_{i}$			(2)
Energy equation	$\frac{𝜕(\rho_{i}C_{i}T_{i})}{𝜕\tau }=\frac{𝜕}{𝜕x_{j}}(\lambda_{i}\frac{𝜕T_{i}}{𝜕x_{j}})+S_{i}\frac{𝜕(\rho_{g}C_{g}T_{g})}{𝜕\tau }+\frac{𝜕(u_{i}\rho_{g}C_{g}T_{g})}{𝜕x_{j}}=\frac{𝜕}{𝜕x_{j}}(\lambda_{g}\frac{𝜕T_{g}}{𝜕x_{j}})+S_{i}$			(3)
Turbulent momentum equation	$\frac{𝜕(\rho_{g}k)}{𝜕\tau }+\frac{𝜕(\rho_{g}ku_{i})}{𝜕x_{i}}=\frac{𝜕}{𝜕x_{j}}\left[\left(\mu +\frac{\rho_{g}c_{\mu }k}{\varepsilon \sigma_{k}}\right)\right]+G_{k}+G_{b}-\rho_{g}\varepsilon -Y_{M}$			(4)
Turbulent energy equation	$\begin{array}{l} \frac{𝜕(\rho_{i}C_{i}T_{i})}{𝜕\tau }=\frac{𝜕}{𝜕x_{j}}(\lambda_{i}\frac{𝜕T_{i}}{𝜕x_{j}})+S_{i}\frac{𝜕(\rho_{g}C_{g}T_{g})}{𝜕\tau }+\frac{𝜕(u_{i}\rho_{g}C_{g}T_{g})}{𝜕x_{j}}\\ =\frac{𝜕}{𝜕x_{j}}(\lambda_{g}\frac{𝜕T_{g}}{𝜕x_{j}})+S_{i}\frac{𝜕(\rho_{g}\varepsilon )}{𝜕\tau }+\frac{𝜕(\rho_{g}\varepsilon u_{i})}{𝜕x_{i}}\\ =\frac{𝜕}{𝜕x_{j}}\left[(\mu +\frac{\rho_{g}C_{\mu }k^{2}}{\varepsilon \sigma_{s}})\frac{𝜕\varepsilon }{𝜕x_{j}}\right]+C_{1\varepsilon }\frac{\varepsilon }{k}C_{3\varepsilon }G_{b}+\rho_{g}C_{1}S\varepsilon -C_{2}\rho_{g}\frac{\varepsilon^{2}}{k+\sqrt{v\varepsilon }} \end{array}$			(5)
P1 model	$q_{r}=-\frac{1}{3(K_{a𝜈}-K_{s𝜈})-{AK}_{s𝜈}}\cdot ▽G$			(6)
	$\frac{𝜕}{𝜕x_{i}}(\Gamma \frac{𝜕G}{𝜕x_{i}})-AG+4AGT^{4}=S_{G}$			(7)
**Symbol**				
ρ	continuous phase density [kg·m^−3^]	$G_{b}$	turbulent kinetic energy from buoyancy [kg⸱m^−1^·s^−1^]	
$S_{m}$	mass source term	Y_M_	fluctuations from over-diffusion [kg⸱m^−1^·s^−1^]	
t	time [h]	$C_{1\varepsilon }$	$C_{1\varepsilon }=max\left(0.43,\frac{\eta }{\eta +5}\right)$	
$\mu_{i}$	gas velocity in the *i* direction [m·s^−1^]	$C_{2}$	1.44	
$\mu_{j}$	gas velocity in the *j* direction [m·s^−1^]	$C_{u}$	1.91	
𝜏_*ij*_	viscous stress [N·m^−3^]	$\sigma_{s}$	0.025	
$F_{i},g_{i}$	external volume force and gravitational volume force in the *i* direction [N⸱m^−3^]	η	$\eta =s\frac{K}{\varepsilon }$	
p	gas pressure [Pa]	$K_{av}$	radiation absorption coefficient	
$\lambda_{g}$	coefficient of thermal conductivity [W⸱m^−1^⸱K^−1^]	$K_{sv}$	scattering coefficient	
$\rho_{g}$	gas density [kg⸱m^−3^]	G	incoming radiation	
$C_{g}$	specific heat capacity of gas [J⸱kg^−1^⸱K^−1^]	A	coefficients of linear each-phase anisotropy phase function	
$T_{g}$	furnace wall temperature [K]	σ	radiation constant, 5.67 × 10^−8^ [W⸱m^−2^⸱K^−4^]	
k	turbulent kinetic energy [m^2^·s^−1^]	$S_{G}$	radiation source term	
ε	dissipation rate of turbulent kinetic energy [m^2^·s^−1^]	$q_{r}$	heat radiation	
$G_{k}$	turbulent kinetic energy generated by laminar velocity gradients [kg⸱m^−1^·s^−1^]	S	positive projection area of fluid and model contact surface [m^2^]	

**Table 2:** Boundary condition parameter settings.

Project	CH_4_ [%]	H_2_ [%]	CO [%]	CO_2_ [%]	Air [%]	Gas Flow [kg/s]	Velocity [m/s]
Combustion chamber	25.5	59.2	6	2.4	6.9	/	5
Carbonization chamber	29	35	10	0.06	25.94	0.6	/
Air inlet 1	/	/	/	/	100	/	12
Air inlet 2	/	/	/	/	100	/	3

**Table 3:** Reference for the selection of silica bricks.

Sample	Young’s Modulus [GPa]	Thermal Expansion Coefficient [10^−6^ °C^−1^]	Thermal Conductivity [W/(m·K)]	Average Thermal Stress [MPa]	Top Temperature of PW [°C]
1	20	10.5	2	14.87	1003.4
2	20	11.0	2.1	16.01	1020.1
3	20	11.5	2.2	17.65	1042.3
4	20	12.0	2.3	19.29	1061.8
5	20	12.5	2.4	21.04	1083.5
6	20	13.0	2.5	22.89	1101.8
7	21	10.5	2	18.32	1003.4
8	21	11.0	2.1	20.13	1020.1
9	21	11.5	2.2	21.76	1042.3
10	21	12.0	2.3	22.82	1061.8
11	21	12.5	2.4	23.84	1083.5
12	21	13.0	2.5	24.88	1101.8
13	22	10.5	2	20.13	1003.4
14	22	11.0	2.1	21.80	1020.1
15	22	11.5	2.2	22.98	1042.3
16	22	12.0	2.3	23.83	1061.8
17	22	12.5	2.4	24.73	1083.5
18	22	13.0	2.5	25.61	1101.8
19	23	10.5	2	26.06	1003.4
20	23	11.0	2.1	21.97	1020.1
21	23	11.5	2.2	23.05	1042.3
22	23	12.0	2.3	24.14	1061.8
23	23	12.5	2.4	24.86	1083.5
24	23	13.0	2.5	26.06	1101.8
25	24	10.5	2	23.18	1003.4
26	24	11.0	2.1	23.76	1020.1
27	24	11.5	2.2	24.73	1042.3
28	24	12.0	2.3	25.61	1061.8
29	24	12.5	2.4	26.63	1083.5
30	24	13.0	2.5	25.35	1101.8
31	25	10.5	2	26.22	1003.4
32	25	11.0	2.1	27.01	1020.1
33	25	11.5	2.2	27.66	1042.3
34	25	12.0	2.3	28.47	1061.8
35	25	12.5	2.4	29.16	1083.5
36	25	13.0	2.5	30.03	1101.8

**Table 4:** Property parameters of different types of silica bricks.

Project		Traditional Silica Brick	Optimized Silica Brick
Young’s modulus [GPa]		22	23
Thermal conductivity [W (m⸱K)]		2.14	2.40
0.2 MPa refractoriness under load [°C]		1669	1681
Thermal expansion coefficient [10^−6^ °C^−1^]	1000 °C	13.4	14.4
	1100 °C	12.1	13.1
	1200 °C	10.9	11.8
	1300 °C	9.2	10.5
	average	11.4	12.5
High-temperature compressive strength [MPa]	1000 °C	26.9	50.1
	1100 °C	18.6	39.9
	1200 °C	16.3	30.7
	1300 °C	15.0	26.0
	average	19.2	36.6

## Data Availability

Data are contained within the article.
